# Diffusion kurtosis imaging detects cortical microstructural alterations in amyloid-positive MCI patients

**DOI:** 10.3389/frdem.2025.1725754

**Published:** 2026-01-06

**Authors:** Rune B. Nielsen, Peter Parbo, Rola Ismail, Rikke B. Dalby, Anna Tietze, Hans Brændgaard, Hanne Gottrup, David J. Brooks, Leif Østergaard, Simon F. Eskildsen

**Affiliations:** 1 Center of Functionally Integrative Neuroscience, Aarhus University, Aarhus, Denmark; 2 Department of Nuclear Medicine, Odense University Hospital, Odense, Denmark; 3 Department of Nuclear Medicine, Vejle Hospital, Vejle, Denmark; 4 Department of Radiology and Nuclear Medicine, Esbjerg and Grindsted Hospital, University Hospital of Southern Denmark, Esbjerg, Denmark; 5 Institute of Neuroradiology, Charité - Universitätsmedizin Berlin, corporate member of Freie Universität Berlin and Humboldt-Universität zu Berlin, Berlin, Germany; 6 Dementia Clinic, Department of Neurology, Aarhus University Hospital, Aarhus, Denmark; 7 Department of Nuclear Medicine and PET Centre, Aarhus University Hospital, Aarhus, Denmark; 8 Institute of Translational and Clinical Research, Newcastle University, Newcastle upon Tyne, United Kingdom; 9 Neuroradiology Research Unit, Aarhus University Hospital, Aarhus, Denmark

**Keywords:** prodromal Alzheimer, mild cognitive impairment, diffusion kurtosis MRI, amyloid PET, MRI, cortex

## Abstract

**Background:**

Alzheimer's disease (AD) is characterized by early accumulation of amyloid-β (Aβ) plaques and tau pathology which precede overt neurodegeneration and cognitive decline. Detecting microstructural brain changes associated with Aβ deposition before the onset of atrophy is critical for early diagnosis and intervention.

**Objective:**

This study investigates whether diffusion kurtosis imaging (DKI) can detect early microstructural alterations in cortical and subcortical gray matter (GM) associated with Aβ pathology in individuals with mild cognitive impairment (MCI).

**Methods:**

Using DKI-derived metrics—mean kurtosis (MK) and mean diffusivity (MD) – we assessed cortical and subcortical microstructure in 67 participants (23 cognitively normal [CN], 44 MCI, including 29 Aβ-positive). Aβ burden was quantified using ^11^C-PiB PET imaging. Cortical atrophy, hippocampal volume, and white matter hyperintensities (WMH) were also evaluated.

**Results:**

Aβ-positive MCI patients exhibited significantly elevated cortical MK, particularly in the left lateral temporal lobe and right precuneus, compared to both CN and Aβ-negative MCI groups. MK positively correlated with Aβ burden in parietal and temporal cortices, even in the absence of cortical atrophy. In contrast, MD showed weaker and less consistent associations with Aβ and was more strongly influenced by age. No significant subcortical MK or MD differences were observed.

**Conclusion:**

Elevated MK in Aβ-positive MCI patients suggests that DKI can detect early microstructural changes associated with the presence of amyloid pathology before the onset of cortical atrophy. MK may serve as a promising non-invasive biomarker for identifying prodromal AD and monitoring disease progression.

## 1 Introduction

The neuropathological features of Alzheimer's disease (AD) include plaques of extracellular cerebral amyloid β (Aβ) protein and intracellular neurofibrillary tangles of hyperphosphorylated tau protein. The accumulation of Aβ is thought to antedate cognitive decline and detectable cerebral atrophy by many years (Jack et al., 2013). Positron emission tomography (PET) offers a means of detecting cerebral fibrillar Aβ and tau tangles several years before cognitive symptoms arise, while magnetic resonance imaging (MRI) is sensitive to brain atrophy in the form of hippocampal volume reduction and cortical thinning (Frisoni et al., 2010). We currently lack tools to non-invasively detect and characterize the cellular injury caused by cerebral protein deposits in the years before overt brain atrophy and clinical AD develops. Such tools are crucial to improve our understanding of AD's preclinical phases and to offer early, preventative therapies to relevant patient cohorts.

Diffusion-weighted MRI (DWI) is sensitive to the micrometer-scale Brownian motions of water molecules and, thereby, to tissue microstructure as these water molecules collide and interact with cell membranes and organelles (Jensen and Helpern, 2010; Jespersen et al., 2010). Indeed, DWI suggests that changes in cerebral microstructure precedes atrophy in subjects with mild cognitive impairment (MCI; Sexton et al., 2011; Struyfs et al., 2015; Weston et al., 2015; Taoka et al., 2016; Gong et al., 2017; Lee et al., 2017; Arab et al., 2018; Gyebnar et al., 2018). By measuring diffusion across multiple directions, it is possible to deduct information about the underlying structural directionality. This technique, dubbed diffusion tensor imaging (DTI), is particularly useful for assessing integrity of white matter (WM) fiber tracts. Nevertheless, in gray matter (GM) where neurotoxic Aβ oligomers and p-tau proteins accumulate in AD, the overall tissue architecture is far more complex with dendrites and axons following less discernible structural patterns. It is feasible to characterize the GM microstructure in terms of the mean diffusivity (MD) under the assumption that water molecules diffuse freely in the tissue. In theory, any microstructure that hinders water-diffusion reduces MD. Conversely, increased MD indicates a less restricted diffusion process potentially associated with neurodegenerative changes such as loss of cells, dendrites, and axons or increased free extracellular water content (Basser and Pierpaoli, 1996; Kale et al., 2006; Weston et al., 2015).

Nonetheless, describing the complex microstructural organization of brain tissue only by a Gaussian model, as in mean diffusivity (MD) from DWI and DTI, remains a considerable oversimplification. Diffusion kurtosis imaging (DKI) addresses this limitation by quantifying the extent to which water diffusion deviates from a Gaussian distribution. The key parameter, mean kurtosis (MK), reflects the average degree of non-Gaussian diffusion and thus serves as an indicator of microstructural complexity. Compared with DTI, DKI requires acquisition at multiple and higher b-values (Jensen and Helpern, 2010). MK may potentially provide additional sensitivity toward early erratic loss of cells, dendrites, and axons in AD, which might not be detected with plain MD (Jensen and Helpern, 2010). Raised MK has been observed in regions with high concentration of Aβ plaques in the transgenic APP/PS1 mouse model (Vanhoutte et al., 2013; Praet et al., 2018). This suggests a role for MK as an early, non-invasive, and relatively cost-effective *in-vivo* biomarker of cellular injury in brain regions associated with neurotoxic amyloid pathology.

In this study, we measured cortical and subcortical gray matter MK and MD using DKI to examine their microstructural changes and the association with Aβ deposition in subjects with MCI.

## 2 Materials and methods

The Regional Ethics Committee for Biomedical Research in the Central Denmark Region approved the study [1-10-72-116-13] in agreement with the declaration of Helsinki, and participants signed an informed written consent ahead of enrolment.

### 2.1 Participants

Subjects with MCI who fulfilled the Petersen criteria (Petersen, 2004) and normal controls (CN) with no cognitive complaints were included in this study. All participants were recruited from national memory clinics and through newspaper advertisements. Please refer to Parbo et al. (2017) for a detailed description of recruitment and inclusion- and exclusion-criteria. We classified participants as Aβ-positive (Aβ+) or Aβ-negative (Aβ-), based on visual reads by experts and a dichotomizing threshold region of interest (ROI) mean ^11^C Pittsburgh Compound B standard uptake value ratio (PiB SUVr) set at 1.5, which naturally separated the bimodal distribution of composite cortical PiB SUVrs into low and high subgroups. In addition, all participants were assessed with MRI and a series of standardized neuropsychological tests recommended for identifying dementia, early cognitive decline and depression, as previously described (Parbo et al., 2017). We present summary scores for Clinical Dementia Rating (CDR), Mini-Mental State Examination (MMSE), and Geriatric Depression Scale (GDS-15). A GDS-15 score of 6 or more was considered an exclusion criterion.

### 2.2 Brain imaging and preprocessing

MRI was acquired on a Siemens Magnetom 3T Skyra system (Siemens Healthcare, Erlangen, Germany) using a 32-channel head coil. DKI was acquired using a b-value scheme optimized for clinical settings, offering both speed and robustness (Hansen et al., 2013, 2014), including one *b* = 0 image, three *b* = 1000 s/mm^2^ and nine *b* = 2500 s/mm^2^ with 2.3 mm isotropic voxels, FOV = 220 × 220 mm^2^ in 38 slices, TR = 12.4 s, TE = 0.107 s and TI = 2.1 s. The duration of the sequence is less than two minutes reducing the risk of movement artifacts. In addition, anatomical T_1_-weighted magnetization-prepared two rapid gradient echo (MP2RAGE; Marques et al., 2010) and T_2_-weighted fluid attenuated inversion recovery (T_2_-FLAIR) MRI were acquired. See Supplementary Material for additional information on MRI sequence settings.

Experienced radiologists screened the anatomical images to ensure exclusion of subjects with structural abnormalities, including tumors, large infarcts, hydrocephalus, and other major structural brain pathology that could significantly impact cognition. Additionally, the degree of chronic small vessel ischemia as indicated by the extent of visible WM hyperintensities (WMH) on T_2_-FLAIR images was graded using the Fazekas scale and quantified by WMH volume segmented using an automatic algorithm (Schmidt et al., 2012).

MD and MK were calculated from DKI with the method described by Hansen and colleagues (Hansen et al., 2013, 2014), using denoised (Veraart et al., 2016) images corrected for Gibbs ringing artifacts (Kellner et al., 2016). To localize cortical and subcortical MD and MK, the DKI were aligned to the MP2RAGE using the *b* = 0 image of the DKI sequence (Avants et al., 2011).

Cerebral amyloid accumulation was characterized visually and as PiB SUVrs, calculated from PiB PET images acquired on a High-Resolution Research Tomograph (ECAT HRRT; CTI/Siemens, Knoxville, TN, USA). As previously described (Parbo et al., 2017), the cerebellar GM mean ROI was used as a non-specific reference. PET images were aligned with corresponding anatomical T_1_-weighted MRIs to localize cortical PiB SUVrs (Collins et al., 1994).

### 2.3 Atrophy, cortical measurements and white matter hyperintensity volume

First, we measured hippocampal volume (Coupe et al., 2011) and cortical thickness to detect atrophy. The latter was defined as the perpendicular distance between the inner and outer cortical borders delineated on MP2RAGE (Eskildsen and Ostergaard, 2006; Aubert-Broche et al., 2013). Then, we extracted cortical values of MK, MD and PiB SUVr at the middle point between these inner and outer cortical borders, using methods described previously (Eskildsen et al., 2017; Nielsen et al., 2017). By sampling along the central cortical layers, partial volume effects were minimized.

WMH volume was measured as the volume of T_2_w-FLAIR WMHs, segmented using a histogram-based method (Smart et al., 2011) followed by region growing and manual inspection and corrections of segmentations. Subject-specific intracranial volume was used for normalization of all volumetric measurements (Eskildsen et al., 2012).

### 2.4 Subcortical diffusion

Cerebral regions involved in the frontal subcortical circuits were automatically segmented, using an atlas (Neuromorphometrics, Inc., Somerville, Massachusetts) located in MNI space. Subcortical regions included bilateral segmentations of the hippocampus, amygdala, thalamus, nucleus accumbens, nucleus caudatus, putamen, and globus pallidus. The atlas was moved into native DKI space for extraction of MK and MD mean ROI values (SPM 12 version 7219, The Wellcome Trust Center for Institute of Neurology, University College London).

### 2.5 Statistical analysis

Statistical computations were performed with R version 4.3 (R Foundation for Statistical Computing, Vienna, Austria). Group differences in cortical and subcortical ROI mean values of MK, MD, and PiB SUVr were assessed using linear regression. Likewise, linear regression was applied to assess correlations between cortical PiB SUVr measurements and cortical MK and MD measurements, respectively. In both cases, we included age as a covariate since aging is known to affect diffusional parameters (Falangola et al., 2008; Gong et al., 2014; Taha et al., 2022). In separate models we additionally adjusted for sex and MMSE scores as some studies have found these to influence diffusion, though mainly in WM (Lawrence et al., 2021; Matsumoto et al., 2025). For the cortical analyses we visualized *t*-values for *p* < 0.05 (one-tailed) and outline clusters surviving family wise error correction (FWE) for multiple comparisons (Worsley et al., 1996) at α = 0.001.

Demographic differences across the three groups of CN subjects, Aβ+ MCI patients and Aβ- MCI patients, respectively, were assessed in a two-step procedure. First, we tested for any differences across all three groups. If any group differences were detected, we compared individual subgroups. For continuous variables following a normal distribution, we assessed differences across all groups using ANOVA, while Tukey's test for *post hoc* multiple pair-wise comparisons interrogated differences between individual groups. Otherwise, we applied the Kruskal–Wallis test across all three groups and *post hoc* Wilcoxon rank-sum test with Bonferroni correction for the individual pairwise group comparisons. Categorical variables were evaluated across all three groups with the extended Fischer's exact test, including *post hoc* pairwise comparisons and Bonferroni correction for comparison of individual groups. We considered *p* < 0.05 (two-tailed) statistically significant.

## 3 Results

Twenty-three CN subjects and 44 MCI patients (29 Aβ+) were included in the study. Their demographic characteristics, neuropsychological test results, WMH volume and hippocampal volume are summarized in Table 1. On average, Aβ+ MCIs were older than Aβ- MCIs, and they scored lower than both Aβ- MCIs and CNs on cognitive tests. Aβ- MCIs performed worse than CNs on cognitive tests with a significantly raised mean CDR sum-of-boxes score. Antihypertensive drug use was more common among Aβ+ MCIs, but all groups showed similar Fazekas scores and WMH volumes, indicating similar vascular lesion load across subjects. We only observed a trend toward reduced hippocampal volume in MCIs compared to CNs. No obvious cortical thinning was detected in any of the MCI groups compared to the CN group (Supplementary Figure S1).

**Table 1:** Demographics, neuropsychological test results, hippocampal volume and WMH load according to subgroup.

**Demographic**	**Aβ+ MCI (*n* = 29)**	**Aβ- MCI (*n* = 15)**	**Cognitively normal (*n* = 23)**	***p*-value**
Age, years, mean ± sd [range]	73.1 ± 6.0 [62–83]	65.6 ± 8.6 [50–79]	68.9 ± 6.6 [58–80]	0.003^a^
Gender, females *n* (%)	9 (31)	8 (53)	14 (61)	0.096
Education, years, median [range]	12.0 [7.0–19.5]	11.5 [7.0–20.0]	13.0 [11.0–19.0]	0.172
Subjects using NSAID, *n* (%)	9 (31)	4 (27)	3 (13)	0.326
Subjects using antihypertensive drugs, *n* (%)	13 (45)	8 (53)	4 (17)	0.040^b^
MMSE score, median [range]	27 [23–30]	28 [23–30]	29 [25–30]	0.001^a, b^
CDR sum of boxes, median [range]	1.5 [0.0–4.0]	1 [0.5–2.0]	0.0 [0.0–0.0]	< 0.001^a, b, c^
Geriatric depression Scale, mean ± sd	1.8 ± 1.9	1.1 ± 1.1	0.39 ± 0.72	0.004^b^
Subjects with Fazekas score 0/1/2/3, *n*	4/13/10/2	5/5/4/1	8/9/5/1	0.236
PiB dose, MBq, mean ± sd (*n*)	371 ± 77	409 ± 26	424 ± 28^*^	0.019^b^
White matter hyperintensity volume, % intracranial volume, median [range]	0.56 [0.02–4.19]	0.25 [0.01–3.39]	0.32 [0.00–4.45]	0.309
Hippocampal volume, mL, mean ± sd	5.13 ± 0.64	5.17 ± 0.92	5.58 ± 0.61	0.055

Demographics presented as mean ± sd, number of subjects (%) or median [range]. *p*-values for group-wise differences were assessed using ANOVA, Kruskal–Wallis test and the extended Fisher's exact test. *Post hoc* pairwise comparisons involved Tukey's test for multiple comparisons, Wilcoxon's rank sum test and Fisher's exact test (Bonferroni corrected). Four cognitively normal controls did not complete the CDR because the required informant was not present. Based on an in-depth interview, their CDR sum of boxes score was set to zero. ^a^*P* < 0.05 for Aβ+ MCI vs. Aβ- MCI; ^b^*P* < 0.05 for Aβ+ MCI vs. controls; ^c^*P* < 0.05 for Aβ- MCI vs. controls. MCI, mild cognitive impairment; CDR, clinical dementia rating; MMSE, mini-mental state examination; NSAID, non-steroidal anti-inflammatory drug; Aβ+, PiB-positive MCI; Aβ-, PiB-negative MCI. ^*^11 cognitively normal controls did not complete an ^11^C-PiB-PET scan and so has undetermined amyloid status.

### 3.1 Mean kurtosis

**Figure 1:**
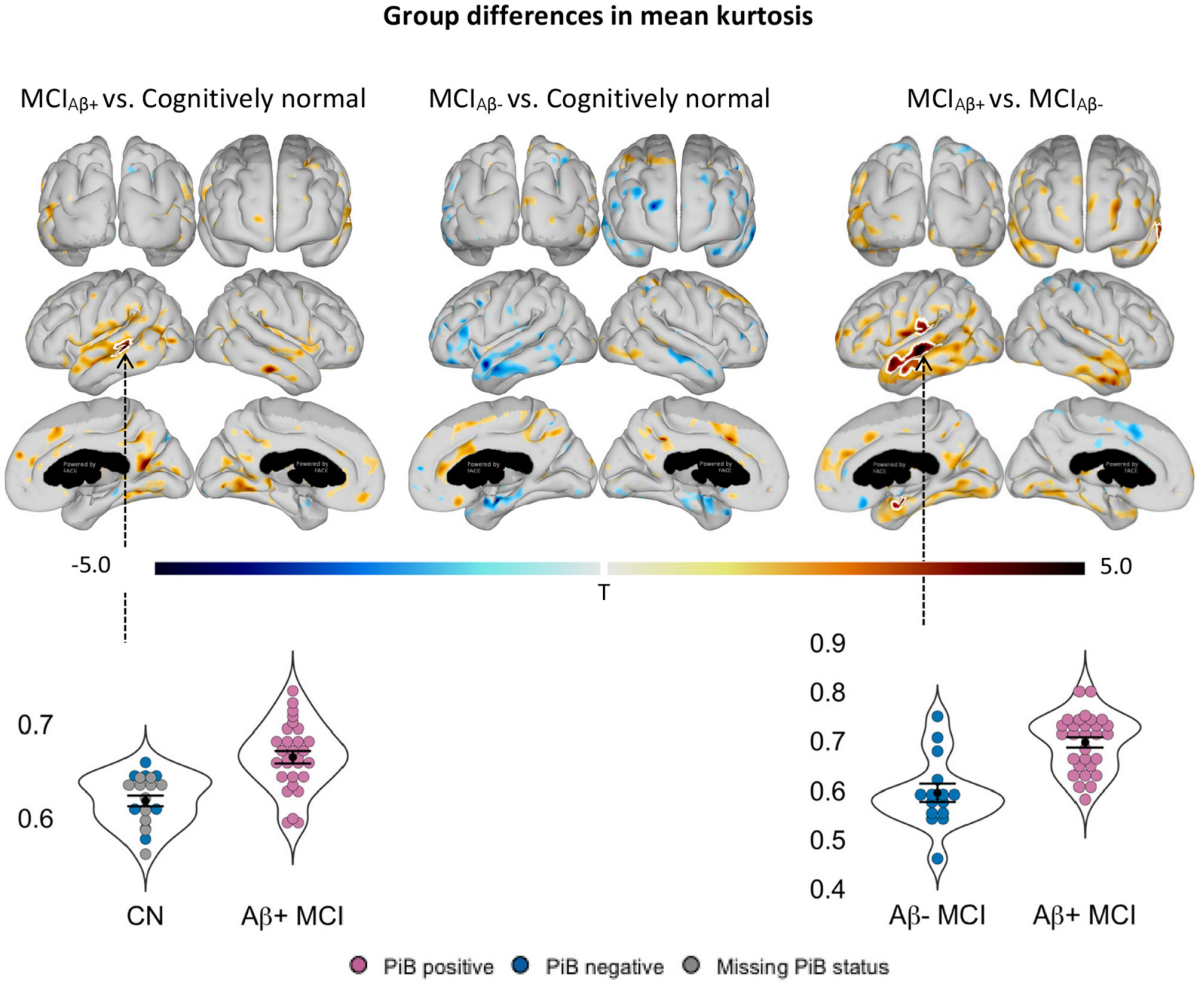
Statistical *t*-maps of significant (*p* < 0.05) differences in cortical mean kurtosis between patients with mild cognitive impairment (MCI) and cognitively normal controls and between subgroups of MCI patients. Negative *t*-values indicate decreases (blue nuances), while positive *t*-values indicate increases (red nuances) in patients. The white outlining marks clusters surviving family-wise error correction for multiple comparisons (*p* < 0.001). Age-adjusted region of interest mean values from clusters with the highest mean *t*-value, as indicated with the black arrows, are shown in the lower panel, including mean (black dot) and standard error of the mean (whiskers). Patients were sub-grouped as amyloid positive (Aβ+) or amyloid negative (Aβ-) based on their ^11^C-PiB (Pittsburgh Compound B) status, defined by an atlas based composite regional ^11^C-PiB SUVr (standard uptake value ratio) >1.5 and ≤ 1.5, respectively. Statistics were adjusted for age using linear regression.

Cortical MK was significantly elevated in the Aβ+ MCI patients compared to the CNs, with clusters surviving FWE correction in parts of the left lateral temporal lobe and in the caudal part of the right precuneus close to the parietooccipital sulcus (Figure 1). The relative changes extended even further across the lateral parts of the left temporal lobe in the Aβ+ MCI patients when compared to the Aβ- MCI patients, indicating an association between Aβ pathology and MK (Figure 1). In further support of this association, MK was trending lower in the Aβ- MCI group compared with the group of CNs which contained some subjects with undetermined Aβ status (Figure 1). Extending the linear models with sex and MMSE only slightly modified the statistical maps without changing the interpretation (Supplementary Figure S3).

**Figure 2:**
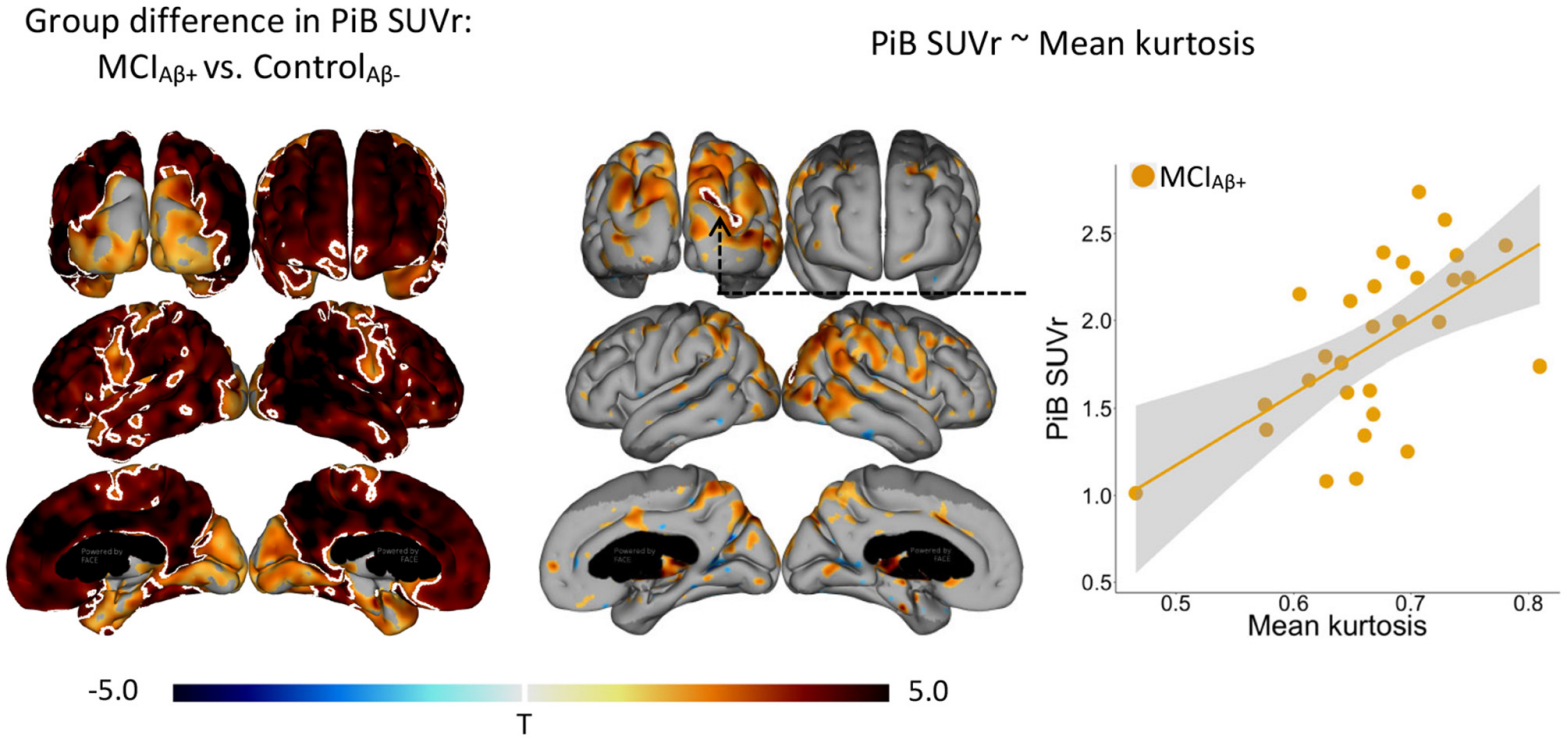
Statistical *t*-value maps showing significant (*p* < 0.05) differences in PiB SUVr and vertex-wise linear correlation between mean kurtosis and PiB SUVr, in amyloid positive (Aβ+) patients with mild cognitive impairment (MCI). Negative *t*-values indicate decrease in Aβ+ MCIs or negative correlation (blue nuances), while positive *t*-values indicate increase in Aβ+ MCIs or positive correlation (red nuances). The white outlining marks clusters surviving family-wise error correction for multiple comparisons (*p* < 0.001). Amyloid status, either positive or amyloid negative, was defined as an atlas based composite regional Pittsburgh compound B (PiB) standard uptake value ratio (SUVR) >1.5 and ≤ 1.5, respectively. The surface-based statistics were adjusted for age using linear regression.

In the Aβ+ MCI group, MK levels and PiB SUVr correlated positively within sub-regions of the parietal, temporal, and occipital cortices (Figure 2). A single cluster in the right parieto-occipital region remained significant after FWE-correction. Notably, these correlations did not overlap with all regions that showed increased cortical MK (Figure 1) or significantly elevated PiB SUVr (Figure 2) in the group of Aβ+ MCI patients. We observed no significant group differences in MK in any of the assessed subcortical structures.

### 3.2 Mean diffusivity

Cortical MD was trending higher in both Aβ+ and Aβ- MCI patients compared to the CN subjects and in Aβ+ MCI patients compared to Aβ- MCI patients (Supplementary Figures S2, S4). While none of the group differences survived the conservative FWE correction for multiple comparisons, the spatial extent of these changes was largest in the group of Aβ+ MCI patients. Cortical levels of MD showed trends toward correlating negatively with overlapping levels of PiB SUVr in Aβ+ MCIs (Supplementary Figure S2). While weak, these correlations could be the result of Aβ accumulation and/or microglial activation limiting diffusion of water in the tissue, thereby reducing MD. The areas of correlation overlapped with areas of MK–PiB SUVr correlations (Figure 2), but were less widespread (Supplementary Figure S2). This differs from the MK findings indicating that separate mechanisms might affect MD in the patients. We found no significant group differences in MD in any of the assessed subcortical structures.

### 3.3 Age effect on diffusion-weighted MRI

**Figure 3:**
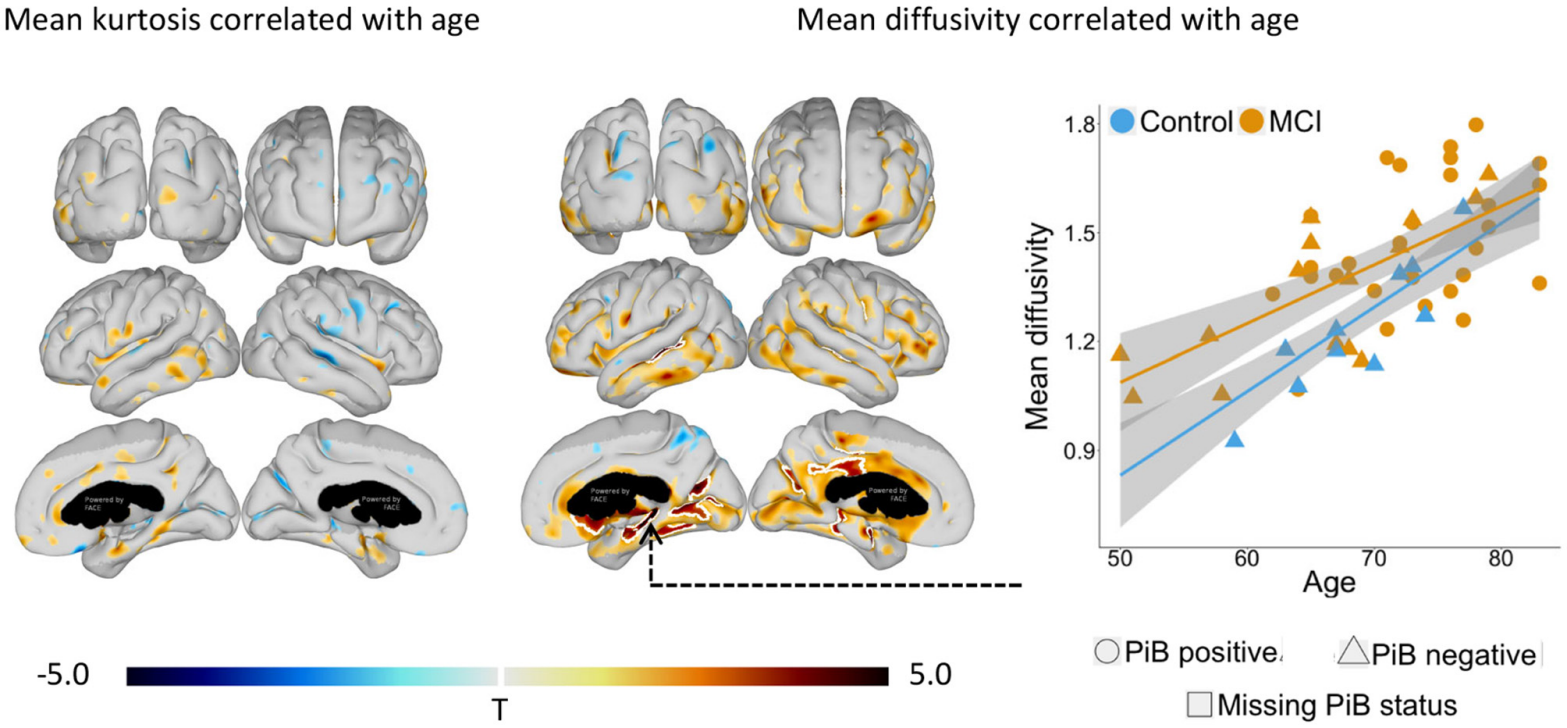
Statistical *t*-value maps showing significant (*p* < 0.05) vertex-wise linear correlation between mean kurtosis or mean diffusivity and age for all subjects. Negative *t-*values indicate negative correlation (blue nuances), while positive *t*-values indicate positive correlation (red nuances). The white outlining marks clusters surviving family-wise error correction for multiple comparisons (*p* < 0.001). PiB (Pittsburgh Compound B) status was defined by an atlas based composite regional 11C-PiB SUVr (standard uptake value ratio) >1.5 and ≤ 1.5, respectively. All correlations were adjusted for study group, either mild cognitive impairment (MCI) or cognitively normal, using linear regression.

Levels of MD correlated positively with subject age within parts of the temporal, cingulate, and occipital cortex (Figure 3). Particularly, strong correlations were observed in the hippocampal area, in the lingual and fusiform gyri, in the subgenual anterior cingulate cortex, and in the parieto-occipital- and lateral temporal lobe. In contrast, we observed no significant correlations between MK and age (Figure 3). Age adjustment was made for all group comparisons.

## 4 Discussion

Using DKI, we show that microstructural changes to the cortex can be observed as elevated MK in patients with prodromal AD before cortical atrophy appears. Secondly, we show that MD, but not MK, increases considerably with subject age, emphasizing the need to adjust for age when evaluating MD.

We suggest that the microstructural changes detected by MK may reflect the toxic effects of Aβ pathology during the prodromal phase of AD, either as a direct effect of Aβ oligomers or an indirect effect of increased neuroinflammation. Consistent with this interpretation, we found significantly higher MK values in Aβ+ MCI patients than in both Aβ- MCI patients and in CN subjects. These elevations were most pronounced in the temporal cortex and precuneus of Aβ+ MCI patients overlapping with regions typically affected by early deposition of Aβ plaques in AD (Lecy et al., 2024). Within these cortical regions, the most prominent hotspots of elevated MK involved parts of the left lateral temporal lobe, and the caudal part of the right precuneus close to the parietooccipital sulcus. In addition, levels of MK correlated positively with levels of ^11^C-PiB SUVr, within sub-regions of the parietal and temporal lobe in the Aβ+ MCIs. The FWE-surviving cluster of significant MK–PiB SUVr correlations was just outside the areas with significant levels of PiB SUVr and did not overlap with areas of significant groups differences in MK. Aβ is known to start aggregating long before symptoms appear and, at the time of diagnosis, amyloid has reached a plateau in many of the primary affected regions (Jack et al., 2013). Thus, correlates of amyloid will be confined to areas where Aβ build up is ongoing. Taken together, our findings indicate that MK may be sensitive to build-up of Aβ aggregates in the tissue (Nam et al., 2025). Within the regions with PiB SUVr–MK correlations, MD was found to be negatively correlated with PiB SUVr strengthening the observation, that aggregation of amyloid leads to restriction of water diffusion. Note that these changes are found in the absence of any significant cortical atrophy, indicating that elevated MK precedes the cortical degenerative changes that develop in parallel with cognitive symptoms in subjects with AD (Jack et al., 2013). In support of our findings, elevated MK has been detected in the cortex and thalamus of transgenic mice with established amyloidosis compared with wild type mice (Vanhoutte et al., 2013). This has been corroborated by observations of greatly increased MK in numerous cortical regions with a high degree of Aβ pathology in the APP/PS1 mice and by a positive correlation between MK and levels of anti-Aβ (clone 4G8) antibody in the same mice (Praet et al., 2018). Similar to the Aβ+ MCI patients examined in our study, the mice did not have established neurodegeneration or major cerebral atrophy, despite having widespread Aβ pathology.

The mechanisms driving the observed increases in MK are unclear. Extracellular Aβ plaques introduce numerous impermeable boundaries into brain tissue. Water molecules diffusing in the extracellular space encounter these plaques, which increase the heterogeneity of diffusion paths and may cause deviations from Gaussian diffusion, captured by kurtosis metrics. Reactive gliosis (astrocytes and microglia) and inflammation occur around plaques. These processes increase cellular density and branching (GFAP-positive astrocytes, IBA1-positive microglia) and add further structural barriers and tortuosity to water diffusion. Praet et al. showed strong correlations between MK and markers of gliosis (GFAP, IBA1) and amyloid load (4G8 staining; Praet et al., 2018).

These findings contrast with other studies, which have addressed DKI in MCI patients with established atrophy and found reductions, rather than increases, in MK. Specifically, reduced MK has been identified in the thalamus, putamen, globus pallidus, and the hippocampus of elderly humans (mean age of 75 years) with late stage MCI (group average MMSE = 23.1; Gong et al., 2017), and in the left hippocampus in a group of relatively less cognitively impaired amnestic MCI patients (group mean MMSE = 26.9) with atrophic hippocampi (Wang et al., 2015). In the latter study, atrophy was detected in the region showing reduced MK, while in the first study, the patients included were at an advanced disease state where extensive brain atrophy is common. The reductions in MK in the MCI patients in these two studies could therefore indicate degenerative changes including loss of cellular boundaries or increased extracellular space, rather than Aβ deposits or neuroinflammation, which were not measured. As MK seemingly initially increases with increasing levels of Aβ (Figure 2; Vanhoutte et al., 2013; Praet et al., 2018), we speculate that these studies might have been biased toward subjects affected by degenerative changes more so than by Aβ deposition. This underlines that MK is in fact sensitive to any microstructural alterations that influence water diffusion in tissue. As MCI progresses toward a clinical AD diagnosis and subsequently advanced stages, neurodegeneration increasingly affects brain regions. On the other hand, the cerebral concentration of Aβ has typically reached a plateau before an AD diagnosis is made (Jack et al., 2013). Because increasing levels of Aβ may elevate MK, while neurodegeneration seemingly reduces MK, MK values may ultimately decrease as the disease advances and neuronal loss worsens. Corroboratively, reduced MK has been observed in patients with moderate to severe AD compared with cognitively normal controls bilaterally in the hippocampus, while mild AD cases showed no MK reductions despite significant atrophy in several brain regions (Wang et al., 2018).

Our results show that MK is a stronger candidate for detection of early microstructural changes in prodromal Alzheimer's disease than MD. Although, elevated cortical MD was observed in both groups of Aβ+ and Aβ- MCI patients compared with cognitively normal controls, MD only weakly correlated with levels of Aβ and in regions overlapping with areas of significant MK–Aβ correlation (Supplementary Figure S2 and Figure 2). Our results point toward changes in MD as mainly an effect of aging (Figure 3), and are thus etiologically non-specific (Muller et al., 2007). The MD changes observed in our study, although weaker, follow in line with previous observations of elevated MD in the inferior temporal lobe of MCI patients (Gyebnar et al., 2018), and in the parietal lobe of MCI patients with more advanced cognitive symptoms (Gong et al., 2017).

In our ROI analysis of hippocampus, amygdala, thalamus, nucleus accumbens, nucleus caudatus, putamen, and globus pallidus, we did not observe any significant subcortical GM changes in either MK or MD between MCI patients and CN subjects. This accords with previous findings that suggest subcortical microstructural changes only emerge at the late stages of MCI and when patients develop clinical AD (Cherubini et al., 2010; Gong et al., 2017). It should be noted that there is a technical limitation to evaluating subcortical microstructural changes with diffusion MRI, since signal-to-noise ratios decrease with distance from the RF coil (Kwok, 2022).

Our interpretations of the data should be taken with caution. First, the limited sample size warrants replications in larger cohorts. Second, the exact mechanism behind the increased MK is unknown. We consider accumulation of proteins and breakdown of membranes as the primary sources of changes to the diffusion parameters measured. However, other factors, such as edema or gliosis, which may originate from a variety of neuropathological conditions, may contribute as well. Third, in almost half of the cognitively normal group amyloid status was not determined with ^11^C-PiB-PET scans. Finally, the sensitivity of the fast DKI sequence can be questioned as only a few diffusion directions are used for fitting the kurtosis model, which may also explain why no subcortical alterations were detected.

Future studies should address whether a DKI sequence with more diffusion directions increases the sensitivity of MK at the expense of increase in scan time. Moreover, a longitudinal study design should be applied to map the temporal trajectory of MK and to address whether MK obtained with fast DKI can be used to identify subjects with prodromal AD.

In conclusion, we found elevated MK in Aβ-positive MCI patients compared to Aβ-negative MCI patients and significant PiB SUVr–MK correlations suggesting that DKI can detect early microstructural changes linked to amyloid pathology before the onset of cortical atrophy.

## Data Availability

The raw data supporting the conclusions of this article is available here: https://doi.org/10.6084/m9.figshare.30850301.
