# Supplementary material for: Diffusion kurtosis imaging detects cortical microstructural alterations in amyloid-positive MCI patients
Source: Front Dement. 2026 Jan 6;4:1725754. doi: 10.3389/frdem.2025.1725754 (PMC12815771; doi:10.3389/frdem.2025.1725754)
Supplement: Supplementary file 1 [file Data_Sheet_1.pdf]

Supplementary material

For the manuscript "Diffusion kurtosis imaging detects cortical microstructural alterations in amyloid-positive MCI patients"

Table S1. MRI acquisition parameters

Sequence	TR (s)	TE (ms)	TI (s)	FOV (mm)	Slices	Voxel size (mm)	Details
T2FLAIR	9	117	2.5	214x221	45	0.7x0.7x3.3	
MP2RAGE	5	2.98	0.7/2.5	240x256	176	1.0x1.0x1.0	Image= $\frac{GRE_{TI1}GRE_{TI2}}{GRE_{TI1}^2 + GRE_{TI2}^2}$
DKI	12.4	107	2.1	220x220	38	2.3x2.3x2.3	1 b=0<br>3 b=1000<br>9 b=2500 s/mm^2

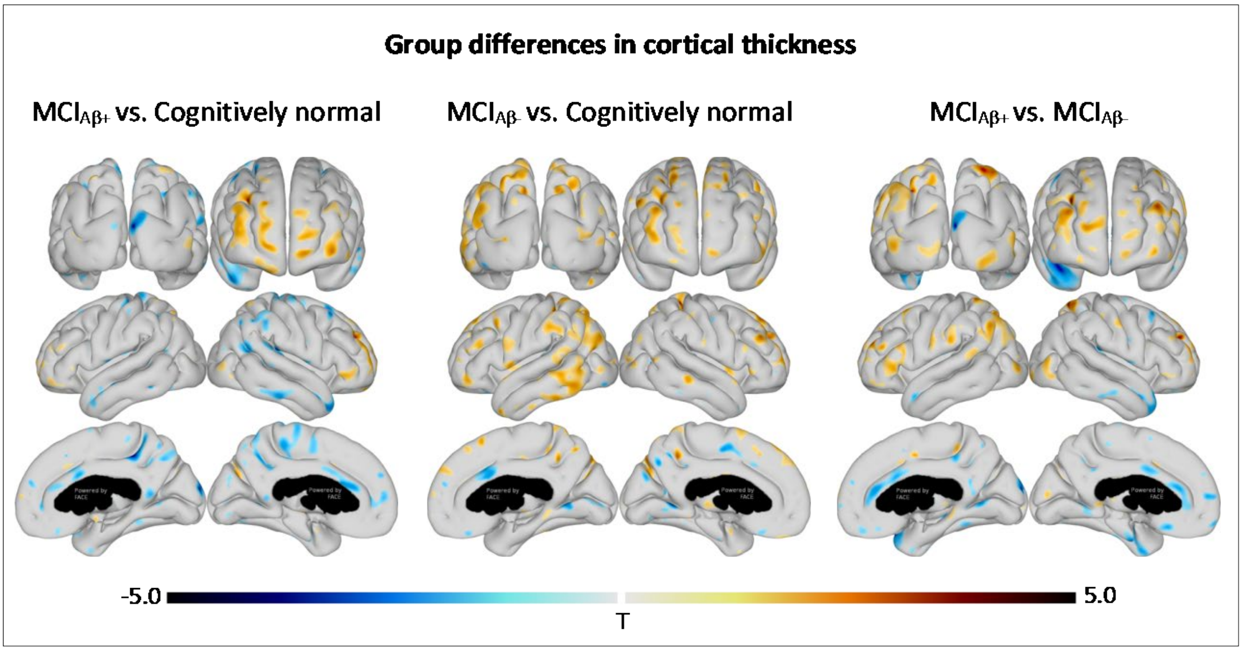

Figure S1. Statistical t-maps of significant ($p<0.05$) differences in cortical thickness between patients with mild cognitive impairment (MCI) and healthy controls and between subgroups of MCI patients. Negative t-values indicate thinner cortex (blue nuances), while positive t-values indicate thicker cortex (red nuances) in patients compared to controls or in amyloid positive (Aβ+) patients compared to amyloid negative (Aβ-) patients. Patients were sub-grouped as Aβ+ or Aβ- based on their PiB (Pittsburgh Compound B) status, defined by an atlas based composite regional PiB SUVR (standard uptake value ratio) >1.5 and ≤1.5, respectively. Statistics were adjusted for age using linear regression. No clusters survived family-wise error correction for multiple comparisons ($p<.001$).

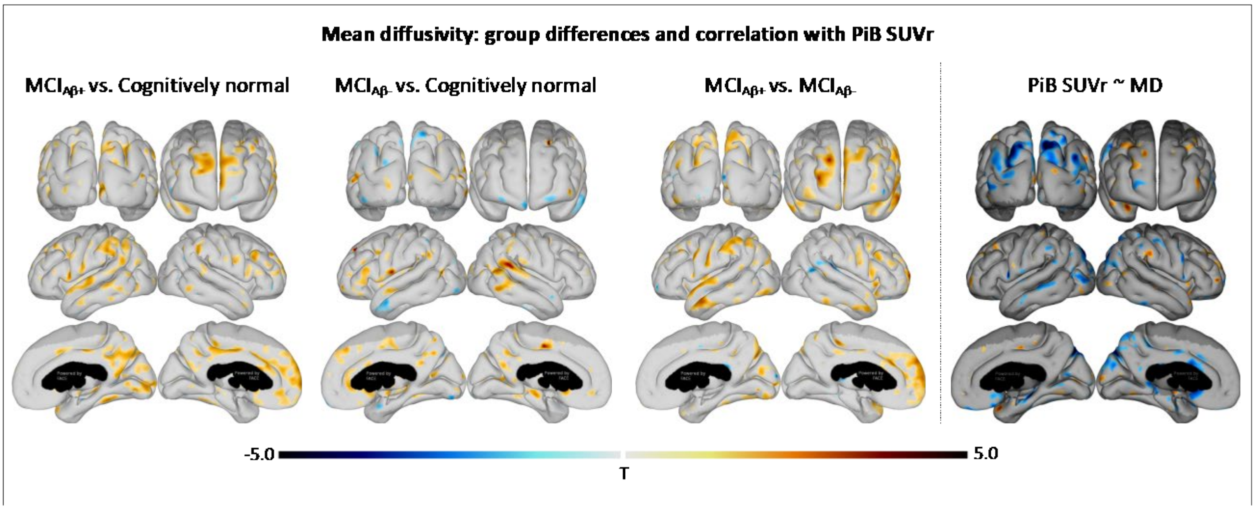

Figure S2. Statistical t-maps showing significant ($p<0.05$) group differences in cortical mean diffusivity and correlation (rightmost panel) between mean diffusivity and PiB SUVR in amyloid positive patients with MCI (mild cognitive impairment). Negative t-values indicate decreases (blue nuances) in MCI patients or negative correlation, while positive t-values indicate increases (red nuances) in MCI patients or positive correlation. No clusters survived family-wise error correction for multiple comparisons ($p<.001$). Amyloid status, either positive (Aβ+) or negative (Aβ-) was defined by an atlas based regional PiB SUVR (standard uptake value ratio) >1.5 and ≤1.5, respectively. Statistics were adjusted for age using linear regression.

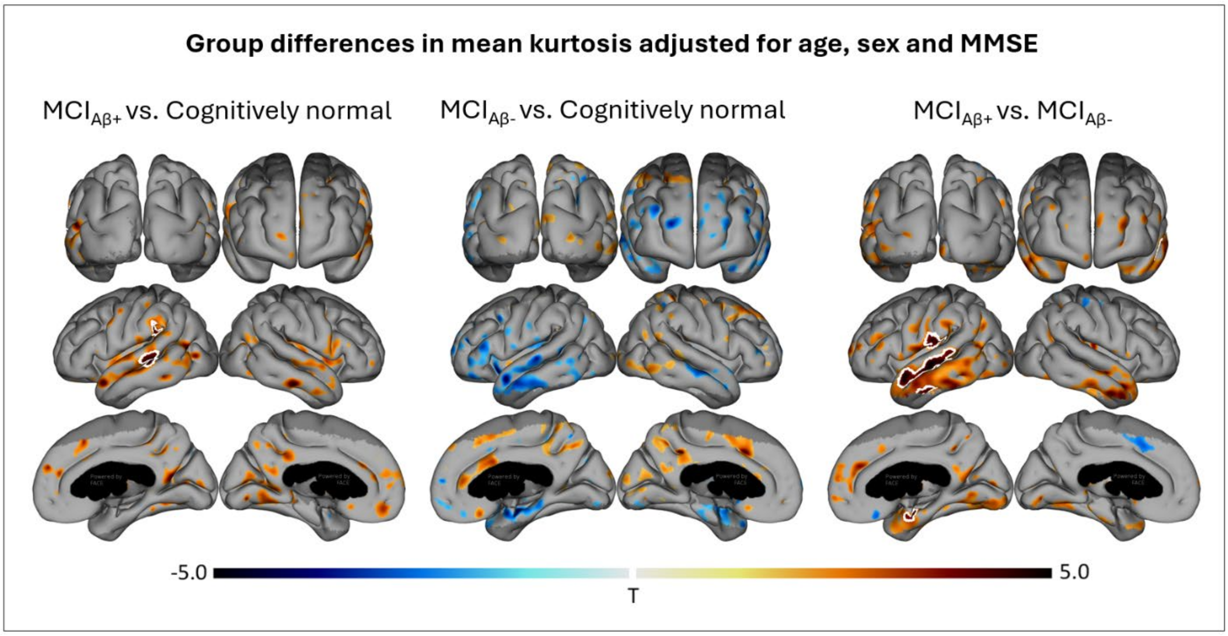

Figure S3. Statistical t-maps of significant ($p<.05$) differences in cortical mean kurtosis between patients with mild cognitive impairment (MCI) and healthy controls and between subgroups of MCI patients. Negative t-values indicate decreases (blue nuances), while positive t-values indicate increases (red nuances) in patients. The white outlining marks clusters surviving family-wise error correction for multiple comparisons ($p<.001$). Patients were sub-grouped as amyloid positive (Aβ+) or amyloid negative (Aβ-) based on their ^{11}C-PiB (Pittsburgh Compound B) status, defined by an atlas based composite regional ^{11}C-PiB SUVr (standard uptake value ratio) >1.5 and ≤1.5, respectively. Statistics were adjusted for age, sex, and mini-mental state examination (MMSE) scores using linear regression.

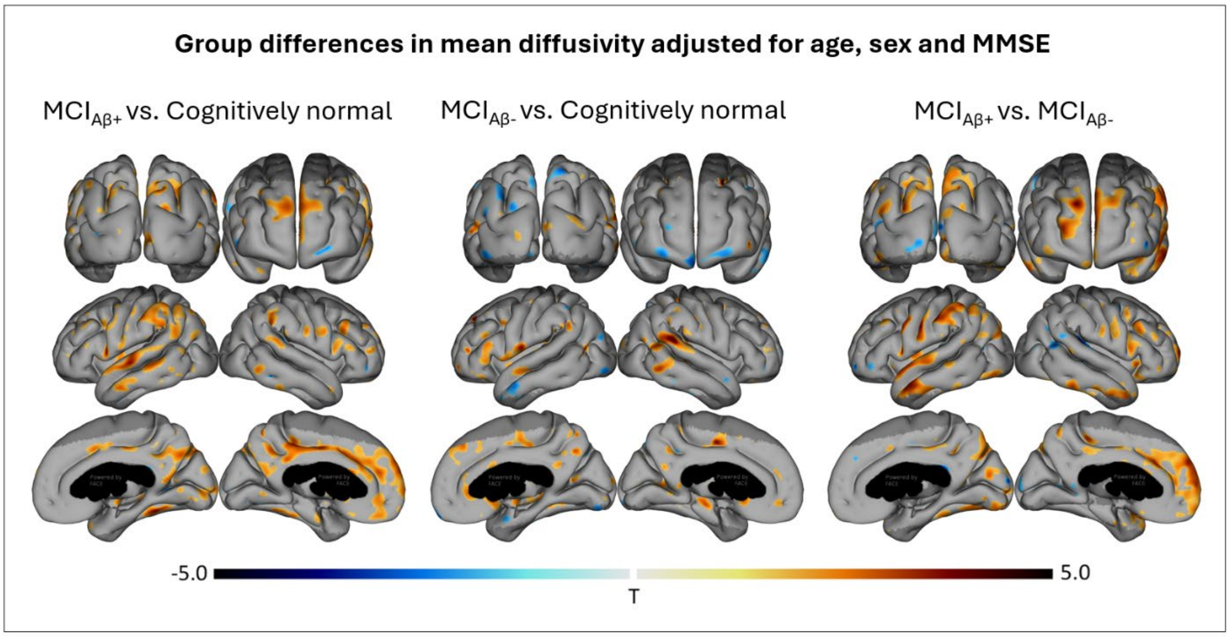

Figure S4. Statistical t-maps of significant ($p<.05$) differences in cortical mean kurtosis between patients with mild cognitive impairment (MCI) and healthy controls and between subgroups of MCI patients. Negative t-values indicate decreases (blue nuances), while positive t-values indicate increases (red nuances) in patients. No clusters survived family-wise error correction for multiple comparisons ($p<.001$). Amyloid status, either positive (Aβ+) or negative (Aβ-) was defined by an atlas based regional PiB SUVR (standard uptake value ratio) >1.5 and ≤1.5, respectively. Statistics were adjusted for age, sex, and mini-mental state examination (MMSE) scores using linear regression.
